# Retroperitoneal Perforation of the Appendix Presenting as a Right Thigh Abscess

**DOI:** 10.1155/2015/707191

**Published:** 2015-03-01

**Authors:** Aditya J. Nanavati, Sanjay Nagral, Nitin Borle

**Affiliations:** Department of General Surgery, K. B. Bhabha Hospital, BandraWest, Mumbai 400050, India

## Abstract

A rare case of a retroperitoneal rupture of the appendix is being reported here. A 53-year-old male presented to us with a right sided thigh abscess. There were not any abdominal complaints at presentation. There was continuous discharge after incision and drainage from the thigh. Isolation, in culture, of an enteric bacterium from the pus prompted an evaluation of the gastrointestinal tract as a possible source. An MRI scan revealed fluid tracking from the right paracolic gutter over the psoas sheath and paraspinal muscle into the thigh. A CT scan revealed the perforation at the base of the appendix into the retroperitoneum. At laparotomy the above findings were confirmed. A segmental ileocaecal resection was done. The patient made an uneventful recovery. The absence of abdominal symptoms at presentation leads to delay in diagnosis in such cases. Nonresolving thigh and groin abscesses should lead to the evaluation of the gastrointestinal tract as origin. Diagnostic clues may also be provided by culture reports what as happened in this case.

## 1. Background

Appendicitis is a very common surgical illness encountered by a surgeon in clinical practice. It has several rare presentations and complications which are widely published. A right thigh abscess due to the retroperitoneal rupture of the appendix is a rare occurrence and has been reported before [1]. In most of the previous reported cases there was either a delayed fecal fistula through the thigh wound [2] or presence of abdominal symptoms along with the thigh abscess that provided diagnostic clues [1]. Diagnosis in the absence of such features is difficult and delayed till the onset of sepsis. We report a case which presented only with a thigh abscess without abdominal symptoms.

## 2. Case Report

A 53-year-old male was admitted with the complaints of pain and swelling in the right thigh for 5 days. There was a low grade fever over this time period. At presentation there were no abdominal symptoms nor was there any backache. Preliminary investigations revealed leukocytosis (total leukocyte count of 15,000/mm^3^) and anemia (haemoglobin 8.5 g/dL). There were warmth and tenderness over the thigh on examination. An ultrasound of the thigh revealed bulky musculature and evidence of pus in the anterior and medial compartment of the thigh. A Doppler study revealed normal vasculature. An incision and drainage was performed and approximately 500 mL pus was drained from the anterior and medial compartments of the thigh. An appropriately collected sample was sent for culture and antibiotic sensitivity.

**Figure 1:**
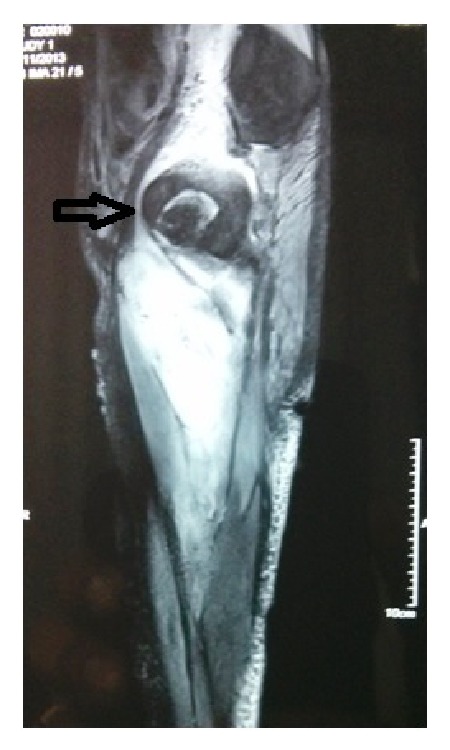
MR STIR image: a sagittal section showing pus tracking from over the psoas under the inguinal ligament into the thigh (arrow).

**Figure 2:**
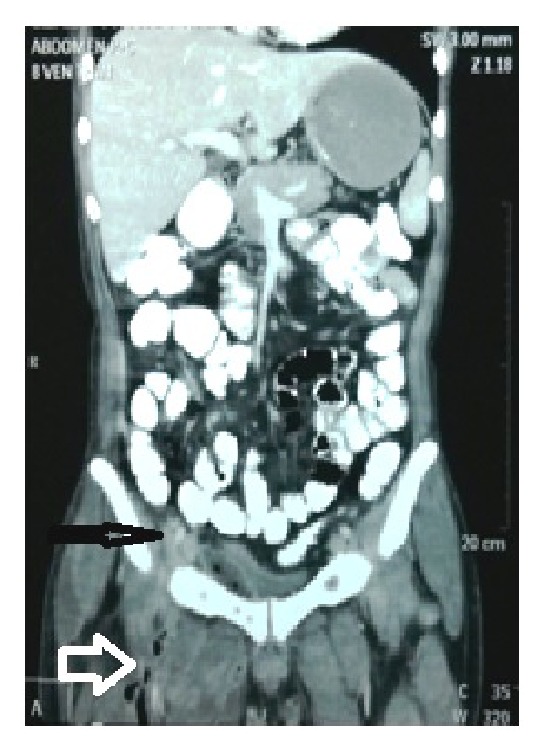
Contrast enhanced CT showing contrast leak (thin black arrow) from the base of the appendix and air specs in soft tissue of the thigh (bold white arrow).

Over the next few days there was continuous discharge of pus from the operative site. The culture report showed growth of* Klebsiella* species which was sensitive to meropenem, amikacin, tigecycline, and colistin. The patient was given meropenem in view of persistent fever and discharge from the thigh. The isolation of an enteric bacterium and continuous pus discharge made us suspect a gastrointestinal source. An MRI of the right thigh with pelvis was hence performed primarily to rule out a perianal source (Figure 1). The MRI revealed the presence of pus in the right paracolic gutter tracking along the right psoas sheath, right quadratus lumborum, and posterior paraspinal muscles via the right lateral part of the pelvic peritoneum into the right gluteal region, right pectineus muscle and the vastus medialis, vastus intermedius, and adductor magnus in the thigh on the right side. A contrast enhanced computerised tomography of the abdomen with oral contrast revealed a small defect in the inferior aspect of the caecum (Figure 2). There was a leak of contrast material into the pericaecal region.

A decision to do an exploratory laparotomy was made. At laparotomy it was discovered that the appendix had completely sloughed off. There was a perforation at the base which was draining into the retroperitoneum. The adjacent caecum was severely inflamed. A decision to do an ileocaecal resection with a double barrel ileocolostomy was made. The retroperitoneum was lavaged and a tube drain inserted in the right paracolic gutter. The patient made an uneventful recovery postoperatively. The drainage through the thigh wound stopped after the laparotomy. At discharge the thigh wound was healing well.

## 3. Discussion

Acute appendicitis is well known to have widely varied presentation some more common and some rather rare. Occasionally some very serious and life-threatening complications may occur in appendicitis. One of the common anatomic variations in the position of the appendix is its retrocaecal position. While in such a position it lay facing the retroperitoneum and the underlying psoas sheath. A perforation with or without gangrene is one of the dreaded complications of appendicitis. A retroperitoneal rupture of an inflamed appendix may not present with classical abdominal symptoms. Such a rupture would classically lead to lower backache or pain at hip flexion due to inflammation of the psoas muscle. The accumulation of pus in the retroperitoneum and/or tracking of such pus along fascial planes into the thigh has also been described [3]. It has been suggested that in unexplained thigh or groin pain with fever and leukocytosis a gastrointestinal source should not be overlooked [4].

Absence of classical symptoms and localizing signs due to the retroperitoneal rupture of the appendix usually results in a delayed diagnosis. Delay in therapy may lead to an increased incidence of complications as well as mortality [5]. Retroperitoneal rupture may present as an appendicular abscess, retroperitoneal abscess, perinephric abscess, or thigh abscess. Some fulminant forms have even presented with abdominal wall sepsis and thigh emphysema [2].

In cases where a thigh abscess has resulted due to appendicitis, the presence of abdominal pain or right flank pain suggestive of appendicitis has prompted investigations like CT scans which make the diagnosis obvious [1, 3]. In cases when abdominal complaints were absent the usual indication of an abdominal source was fecal matter draining through the incision site [2]. In our case there was complete absence of abdominal symptoms from the outset as well as no fecal drainage. At incision and drainage the thigh muscles were normal but an extensive amount of pus had been drained. The culture report showed growth of* Klebsiella* species. The patient was started on appropriate antibiotics but what was surprising was the fact that the culture grew an enteric bacterium in an apparently spontaneous thigh abscess. The patient gave no history of trauma to the thigh. This prompted us to investigate the gastrointestinal tract as the site of origin. We performed an MRI scan as our initial thought was to rule out anorectal pathology. The following CT scan eventually led to the discovery that a perforated sloughed-off appendix was the cause of the abscess. There is merit in associating the bacteria localised from an unusual site to the site where it is known to be found. As more often than not such a finding suggests an unnatural communication between the two sites.

## 4. Conclusion

It is critical that in a case of unexplained thigh and groin abscesses the gastrointestinal system is evaluated as a potential source. Diagnostic clues may be obtained from radiologic investigations like CT or MRI. Microbiologic studies too may provide important information if the bacteriology is appropriately interpreted. It is important to make an early diagnosis and offer timely treatment as delay is associated with higher morbidity.
